# Geriatric nutritional risk index and controller nutritional status score before metastatic first-line chemotherapy predict survival in patients over 70 years of age with metastatic bladder cancer

**DOI:** 10.3389/fmed.2024.1376607

**Published:** 2024-05-10

**Authors:** Onur Yazdan Balçık, Bilgin Demir, Yusuf Ilhan, Baran Akagündüz

**Affiliations:** ^1^Department of Medical Oncology, Mardin Training and Research Hospital, Mardin, Türkiye; ^2^Department of Medical Oncology, Ataturk State Hospital, Aydın, Türkiye; ^3^Department of Medical Oncology, Antalya Training and Research Hospital, Antalya, Türkiye; ^4^Department of Medical Oncology, Mengücek Gazi Training and Research Hospital, Erzincan, Türkiye

**Keywords:** GNRI, CONUT, geriatric, bladder cancer overall survival, nutrition, progression

## Abstract

**Introduction:**

Several prognostic factors have been identified in patients with metastatic bladder cancer (BC). As it is known, older adult patients are prone to nutritional deficiency. The knowledge about nutrition and impact on survival in older patients with metastatic bladder cancer is missing. It is necessary to specifically examine this population. Because timely interventions can make a positive impact on this patients population. This retrospective study aimed to evaluate the prognostic effect of the Geriatric Nutritional Risk Index (GNRI), Controller Nutritional Status (CONUT) score and Prognostic Nutritional Index (PNI) before first-line chemotherapy in the metastatic stage in patients with metastatic bladder cancer over 70.

**Participants and methods:**

Patients over 70 with pathologically confirmed denovo metastatic or recurrent metastatic bladder cancer were included in the study. Patients with infections diagnosed at the time of diagnosis, autoimmune diseases or history of steroid use were excluded. Since our population consists of a specific age group with a specific cancer, we found a new cut-off value by performing ROC analysis to ensure optimal sensitivity and specificity in terms of progression. Low GNRI value was related with poor nutritional status. Low PNI value was related with poor nutritional status and high CONUT score was related with poor nutritional status. Factors predicting overall survival (OS) and Progression-Free Survival (PFS) were assessed using both univariate and multivariate Cox proportional hazards analyses.

**Results:**

106 patients were included in the study and the average age was 75.5 years. In the GNRI-Low group, PFS was significantly shorter than that in the GNRI-High group [HR (95% CI) = 57.1 (12.8–255.5), (*p* < 0.001)]. Among those with a low-CONUT score, PFS was found to be longer than that in the high-CONUT group [HR (95% CI) = 1.7 (1.0–3.0), (*p* = 0.039)]. The median PFS of the PNI-Low group wasn’t significantly shorter than that of the PNI-High group [HR (95% CI) = 1.8 (0.5–6.2), (*p* = 0.359)].

**Conclusion:**

Our study suggests that the GNRI and CONUT scores are useful for predicting survival in patients over 70 years of age with BC.

## 1 Introduction

Bladder cancer (BC) ranks 11th among cancers worldwide. It is four times more common in men than in women. About 10–15% of patients are metastatic at the time of diagnosis (1, 2). Although immunotherapy and other drug conjugates slightly improve prognosis in metastatic BC, platinum regimens have remained the standard of care since the early 1980s, with an overall survival (OS) of approximately 15 months (3, 4). Age, performance status, renal function, and sites of metastasis have been recognized as key prognostic factors in metastatic bladder cancer (BC) (5, 6). Determining reversible nutritional prognostic factors that may have an impact on prognosis, especially in frail and older patients is important. Because timely interventions can make a positive impact on this patient’s population. Hence, there is a necessity to delineate additional prognostic factors linked to survival in BC patients.

Malnutrition is prevalent among older individuals diagnosed with cancer, often stemming from reduced food intake, altered nutrient processing, and the impact of cancer therapies. This condition not only affects treatment decisions but also escalates the likelihood of complications and mortality, prolongs hospitalizations, and diminishes overall quality of life (7, 8). The significance of nutritional status is on the rise among cancer patients. Cancer-related cachexia often leads to muscle wasting and protein depletion, triggering systemic inflammation and hypercatabolism. Malnourished patients experience heightened toxicity rates, reduced treatment compliance, and adverse impacts on survival (9, 10).

Many methods such as Subjective Global Assessment (SGA) score and Mini-Nutritional Assessment-Short Form (MNA-SF) score (11), HALP score (12) modified Glasgow prognostic score (13) have been used to evaluate bladder cancer prognosis.

There is a need for prognostic factors that can be an objective evaluation tool, independent of physical measurements, history and anamnesis taken from the patient, which can be calculated in a short time, are non-invasive and do not require any special skills (14, 15). Nutritional status is related with survival and quality of life in older patients with cancer. We need simple blood-based biomarkers to evaluate malnutrition to predict survival and intervention. Therefore, we decided to evaluate prognostic indexes such as Geriatric Nutritional Risk Index (GNRI), Control of Nutritional Status (CONUT), Prognostic Nutritional index (PNI) in metastatic bladder cancer.

GNRI is a screening tool to predict nutrition-related risk of morbidity and mortality in older patients. GNRI is calculated using body weight, height, and serum albumin levels (16). A low GNRI has been associated with poor prognosis in many cancer types, such as prostate cancer and non-small cell lung cancer (NSCLC) (17, 18). Low GNRI has been shown to be associated with a significantly increased risk of 1-month mortality after BC surgery (19).

CONUT score is comprising serum albumin, total cholesterol, and total lymphocyte counts (20). A high CONUT score has been associated with poor prognosis (21). In a meta-analysis of esophageal and gastric cancer patients comprised the majority, a high CONUT score was associated with poor prognosis (22). In BC patients several studies demonstrated that high CONUT score was associated with poor prognosis (23).

PNI was calculated using albumin and lymphocyte counts (24). Low PNI has been found to be poor prognostic in small cell lung cancer (SCLC) and metastatic laryngeal cancer. In addition, a low PNI was found to have a poor prognosis in a study of patients with geriatric non-Hodgkin lymphoma (25, 26). A meta-analysis evaluating 13 studies with bladder cancer showed that low PNI was associated with worse survival (23).

To our knowledge, there is no other study evaluating GNRI CONUT, and PNI scores in patients over 70 years of age with metastatic BC.

This retrospective study aimed to determine whether CONUT score, GNRI, and PNI before metastatic first-line chemotherapy could be used as useful indicators of survival in metastatic BC patients.

## 2 Participants and methods

Patients with pathologically diagnosed metastatic BC aged 70 years and over in three different centers in Turkey between May 2016 and December 2022 were included in this study. We divided the patients into two groups; Patients who are metastatic at the time of diagnosis (denovo) or patients who are not metastatic at the time of diagnosis but are followed with surgery and/or adjuvant treatments and subsequently develop recurrence (recurrent metastatic).

Patient records and hospital databases were retrospectively reviewed. Demographic characteristics, parameters such as height and weight, initial hemogram parameters (neutrophil, monocyte, and lymphocyte counts), and biochemical parameters (albumin and total cholesterol) were recorded. The death dates of the patients who died were accessed from the death notification system where death records are kept in Turkey. The last arrival date of the surviving patients was accessed from the database of 3 different hospitals and survival outcomes were documented. Patients with a history of other malignant tumors, steroid use, known autoimmune disease, active infection at the time of diagnosis, extra-bladder urothelial disease, or missing data were excluded from the study.

GNRI, CONUT and PNI scores were determined according to the basic hematological and biochemical parameters of denovo metastatic patients before receiving their first-line chemotherapy, while recurrent metastatic patients were determined according to the parameters before receiving their first-line chemotherapy after the development of metastasis. The GNRI was computed using the formula: [1.489 × albumin g/L] + [41.7 × body weight/ideal body weight]. Ideal body weight was calculated as: body height^2^(m) x 22. If body weight/ideal body weight is greater than 1, this ratio is taken as 1. Since our population consists of a specific age group with a specific cancer, we found a new cut-off value by performing ROC analysis to ensure optimal sensitivity and specificity in terms of progression. The Controlled Nutritional Status (CONUT) score was derived from total cholesterol level, total lymphocyte count, and peripheral albumin level (19). PNI was calculated as 10 × serum albumin value (g/dl) + 0.005 × peripheral lymphocyte count (per mm3) (27). Low GNRI value was related with poor nutritional status. Low PNI value was related with poor nutritional status and high CONUT score was related with poor nutritional status.

The analysis was performed using SPSS 28.0 software. OS was defined as the time from diagnosis to death or last visit for denovo metastatic patients, while for recurrent metastatic patients it was defined as the time from the date of metastasis to death or last visit. PFS was calculated as the time from the date of metastatic diagnosis to the date of progression or date of last observation. Patients were considered censored on the date of the last follow-up visit.

### 2.1 Statistical analysis

Data were summarized using descriptive statistics such as mean, standard deviation, median, minimum, maximum, frequency, and ratios. The distribution of variables was assessed using the Kolmogorov–Smirnov test. Independent quantitative data were analyzed using independent samples t-tests and Mann–Whitney U tests. Chi-square test was employed for qualitative independent data, with the Fischer test used when conditions for the chi-square test were not met. A significant cut-off point was observed, and sensitivity, specificity, and positive and negative predictive values were detected. Survival analyzes of prognostic indices and clinical and pathological features were calculated using the Kaplan–Meier method (log-rank test). Multivariate analyzes were used to identify independent prognostic variables based on the stepwise Cox proportional hazards regression model and variables potentially affecting survival (*p* < 0.05 in univariate analyses). The relationship between survival time and each independent factor was indicated using the 95% confidence interval (CI). Statistical significance was set at *p* < 0.05.

This study was planned and conducted in accordance with Good Clinical Practices and the Declaration of Helsinki and was approved by the ethics committee of Diyarbakır Gazi Yaşargil Training and Research Hospital (date of approval and no:23.6.2023–448).

## 3 Results

### 3.1 Optimal cutoff values of geriatric nutritional risk index, controller nutritional status score and prognostic nutritional index

**Figure 1:**
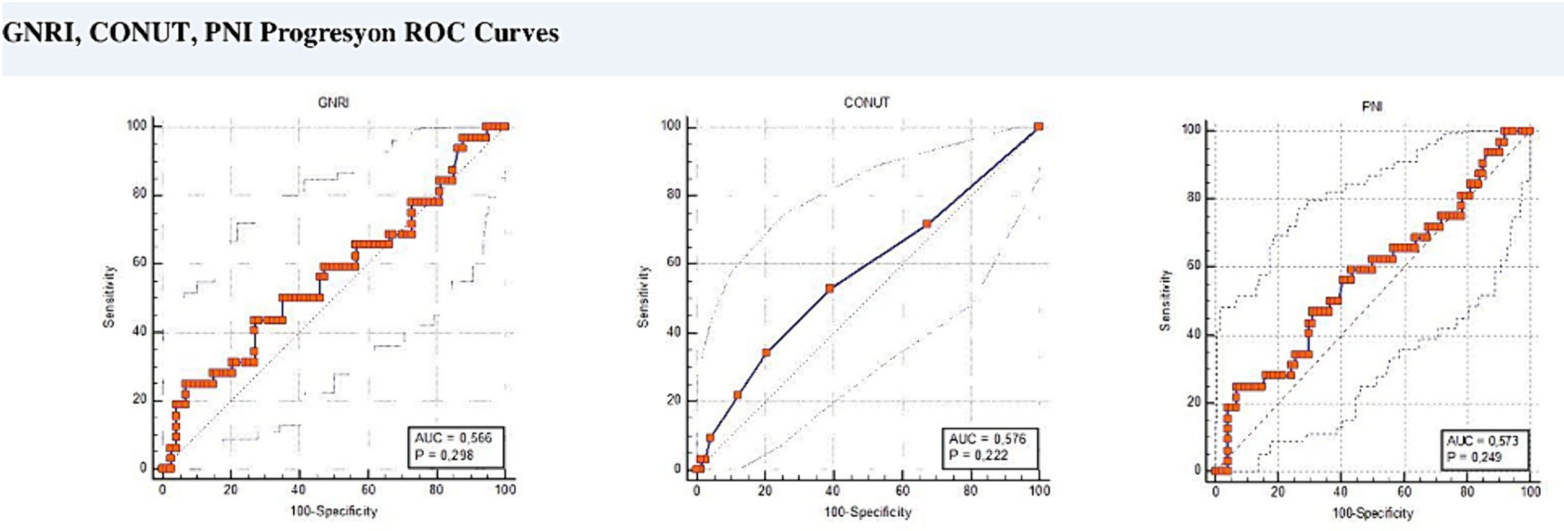
ROC curves for cut-off values.

ROC analysis was performed to determine the most optimal value for progression. This cut-off value was found to be 54 for GNRI (AUC = 0.7; 95% CI 0.6–0.7, *p* < 0.008, sensitivity 91%, specificity 39%). If GNRI ≤54, it was classified as GNRI-Low, and if >54, it was classified as GNRI-High. This cut-off value was found to be 2 for the CONUT score (AUC = 0.6; 95% CI 0.5–0.7, *p* < 0.019, sensitivity 94, specificity 33%). When the CONUT score was ≤2, it was classified as CONUT-Low, and when >2 it was classified as CONUT-High. The optimal cut-off value for PNI was found to be 37 (AUC = 0.7; 95% CI 0.6–0.7, *p* < 0.005, sensitivity 94, specificity 32%). PNI score was classified as PNI-Low when ≤37 and PNI-High when >37 (Figure 1).

### 3.2 Population characteristics

A total of 106 patients were included in this study and the median age was calculated as 75.5 (71–79) years. Of these patients, 89 (84.0%) were male. The number of patients with an Eastern Cooperative Oncology Group (ECOG) performance scale score of 0 or 1 was 79 (74%). While 62 (58.49%) of our patients were denovo metastatic, 44 (41.51%) were recurrent metastatic. The average time from diagnosis to metastasis development in recurrent metastatic patients is 4.7 years. The number of patients receiving TUR-M (Transurethral resection) and intravesical BCG as local treatment was 24 (22.4%). Patients received intravesical chemotherapy for 6 months. Adjuvant radiotherapy was received by 26 (24.5%) individuals. Patients received adjuvant radiotherapy for 10 workdays. The number of patients who underwent cystectomy surgery was 13 (12.3%). These 13 patients received adjuvant chemotherapy. They received adjuvant chemotherapy with a platinum analog (carboplatin or cisplatin) plus gemcitabine on the first day and gemcitabine alone on the 8th day, for a total of 3 months. The most common site of metastasis was the lung with 43.4% (46 patients). Of the total patients, 67% (*n* = 71) died. Demographic information of all patients is presented in Table 1.

**Table 1:** Baseline characteristics of the all population.

		n	%
Gender	Male	89	84,0%
	Female	17	16,0%
BMI	<18.5	6	5,7%
	≥18.5	100	94,3%
Smoking	No	19	17,9%
	Yes	20	18,9%
	Ex smoker	67	63,2%
DISEASE STATUS			
Denovo Metastatic		62	58,5%
Recurrent Metastatic		44	41,5%
TUR-M and B-HCG	No	87	82,4%
	Yes	19	17,6%
Adjuvant RT	No	80	75,5%
	Yes	26	24,5%
Adjuvant CT	No	93	87,7%
	Yes	13	12,3%
HT	No	41	38,7%
	Yes	65	61,3%
DM	No	80	75,5%
	Yes	26	24,5%
ECOG	<2	79	74,5%
	≥2	25	25.5
Liver Metastasis	No	91	85,9%
	Yes	15	14,2%
Lung Metastasis	No	60	56,6%
	Yes	46	43,4%
Bone Metastasis	No	79	74,5%
	Yes	27	25,5%
Exitus	No	20	18,9%
	Yes	86	81,1%

BMI, body mass index; CT, Chemotherapy; DM, diabetes mellitus; HT, hypertension; RT, radiotherapy; TUR, Transurethral resection.

### 3.3 Characteristics of groups according to univariate analysis

Number of GNRI-High group patients is 63, the number of GNRI-Low group patients is 43. In the GNRI-High group, the proportion of male patients was significantly higher than that in the GNRI-Low group (*p* < 0.05). Likewise, the ECOG 0–1 score in the GNRI-High group was significantly higher than in the group with GNRI-Low group (*p* < 0.05; Supplementary Table 1).

Number of CONUT-High group patients is 26, the number of CONUT-Low group patients is 80. The number of patients with ECOG scores II-III in the CONUT-Low group was significantly higher than in the CONUT- High group (*p* < 0.05; Supplementary Table 2).

Number of PNI-High group patients is 66, the number of PNI-Low group patients is 40. The proportion of male patients was significantly higher in the PNI-High group than in the PNI-Low group (*p* < 0.05). Similarly, the ECOG 0- I score in the PNI-High group was significantly higher than that in the PNI-Low group (*p* < 0.05). The exit us rate in the PNI-high group was significantly higher than that in the PNI-low group (*p* < 0.05). Apart from this, no statistically significant difference was found between the PNI-High and PNI-Low groups (*p* > 0.05; Supplementary Table 3).

### 3.4 Progression-free survival analyzes

**Figure 2:**
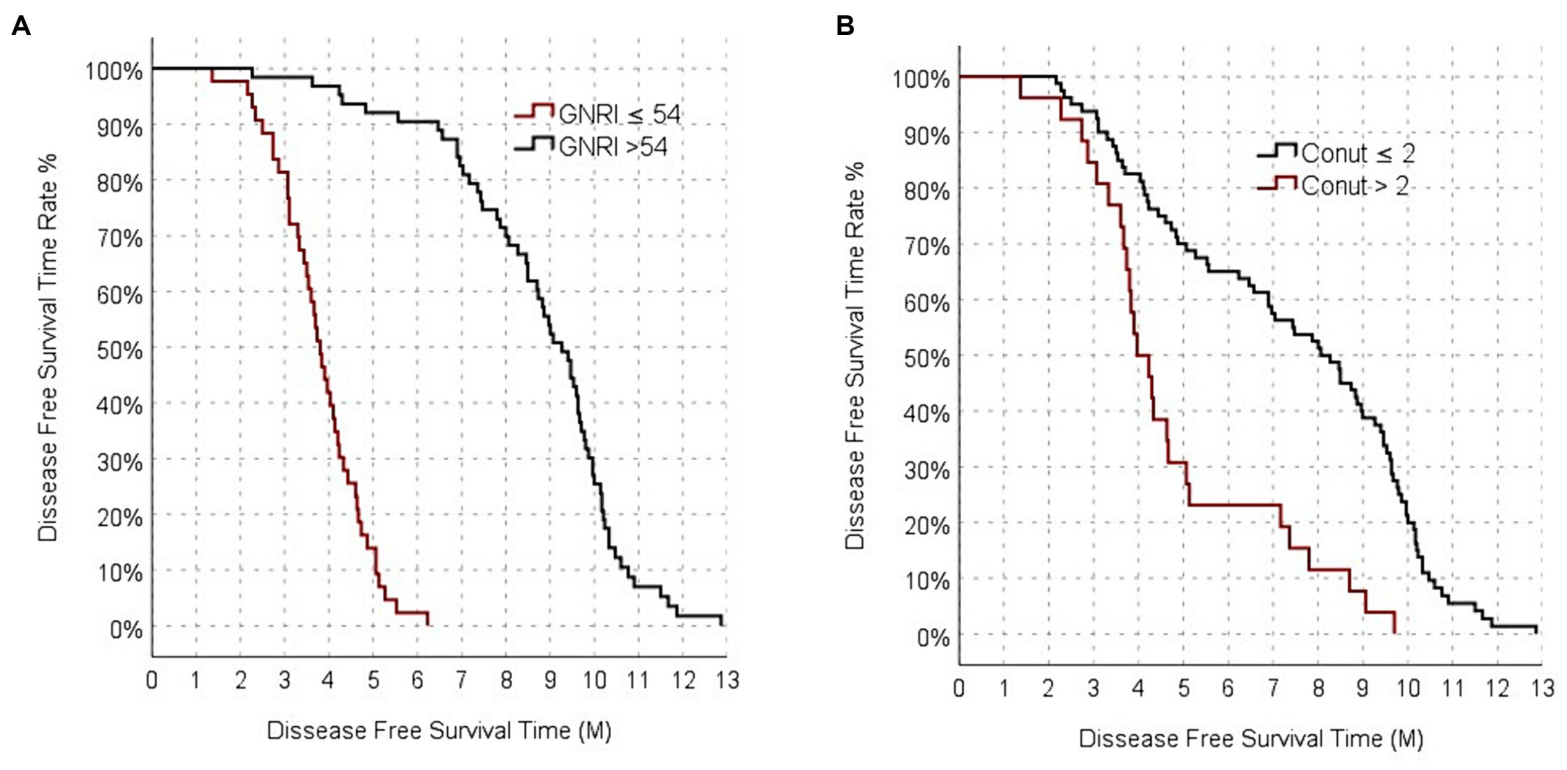
Progression-free survival analysis functions between GNRI **(A)**, and CONUT **(B)**.

The median PFS of all patients in the study was 6.9 (95% CI: 5.3–8.5) months. The median PFS of the GNRI-High group was 9.27 (95% CI:8.6–9.93) months, while the median PFS of the GNRI-Low group was 3.8 (95% CI:3.5–4.1) months. The median PFS of the GNRI-High group was significantly longer than that of the GNRI-Low group (*p* < 0.001; Figure 2).

The median PFS was 8.5 (95% CI: 7.5–9.1) months for CONUT-Low group, it was 4.3 (95% CI: 3.9–4.7) months for the CONUT- High group. The median PFS of the CONUT-Low group was significantly longer than that of the CONUT- High group (*p* < 0.001; Figure 2).

The median PFS of the PNI-High group was 9 (95% CI:8.4–9.7) months, while the median PFS of the PNI-Low group was calculated as 3.80 (95% CI: 3.4–4.2) months. The median PFS of the PNI-Low group was significantly shorter than that of the PNI-High group (*p* < 0.001).

The median PFS was 5.1 (95% CI: 3.7–6.5) months in male patients. It was 7.4 (95% CI: 6.1–8.7) months in female patients, but it was not statistically significant (*p*: 0.337). The median PFS of those with ECOG Performance score I was 8 (95% CI: 6.7–9.3) months, while the median PFS of those with ECOG II-III was 4.3 (95% CI: 3.8–4.9) months. It was statistically significant (*p*: 0.004). While the median PFS of those receiving platinum plus taxane as metastatic first-line chemotherapy was 7.4 (95% CI: 6.1–8.7) months, the median PFS of those receiving single-agent gemcitabine was 4.2 (95% CI: 1.1–7.4) months. The median PFS of patients receiving the platinum plus gemcitabine combination was found to be 3.5 (95% CI: 2.3–4.1) months. The median PFS of those receiving platinum plus taxane was statistically higher and more significant than the other groups (*p*: 0.008). No significance was found in median PFS according to lung, liver, and bone metastases. No significance was found in median PFS according to diabetes mellitus and hypertension. PFS according to univariate analysis is shown in Table 2.

**Table 2:** Univariate and multivariate analysis of PFS.

		Univariate	% 95 HR		*p*	Multivariate	% 95 HR	*p*
Overall		6.9	5.3	8.5				
Gender	Female	7.4	6.1	8.8	0.337			
	Male	5.1	3.7	6.6				
ECOG	≤2	8.0	6.7	9.3	**0.004**	Ref		0.63
	>2	4.3	3.8	4.9		1.1 (0.7–1.9)		
BMI (Kg/m2)	<18.5	9.0	1.0	16.9	0.431			
	18.5–25	5.6	3.1	8.1				
	>25	7.0	5.5	8.4				
First-Line CT								
Platinum + Gemcitabine		3.5	2.3	4.8	**0.008**			
Platinum + Taxane		7.4	6.1	8.7				
Gemcitabine		4.2	1.1	7.4				
Liver metastasis	Yes	6.2	4.4	8.0	0.364			
	No	8.5	7.2	9.9				
Lung metastasis	Yes	5.6	2.3	8.9	0.440			
	No	6.9	6.1	7.7				
Bone metastasis	Yes	7.0	5.1	8.8	0.632			
	No	6.5	3.2	9.7				
HT	Yes	7.8	4.1	11.5	0.351			
	No	6.9	4.8	9.0				
DM	Yes	6.9	4.9	9.0	0.346			
	No	6.5	3.2	9.7				
GNRI	≤54	3.8	3.5	4.1	**<0.001**	57.1 (12.8–255.5)		**<0.001**
	>54	9.3	8.6	9.9		Ref		
PNI	≤37	3.8	3.4	4.12	**<0.001**	1.8 (0.5–6.2)		0.359
	>37	9.0	8.4	9.7		Ref		
CONUT	<2	8.5	7.5	9.5	**<0.001**	Ref		**0.039**
	≥2	4.7	3.9	4.3		1.8 (1–3)		

BMI, body mass index; CT, Chemotherapy; CONUT, controlling nutritional status; DM, diabetes mellitus; GNRI, geriatric nutritional risk index; HR, hazard ratio; HT, hypertension; PFS, progression-free survival; PNI, prognostic nutritional index; RT, radiotherapy.

According to the univariate analysis results, there was significance in GNRI, PNI, CONUT score, first-line chemotherapy selection, ECOG, and gender parameters. Since our primary hypothesis was to examine the effect of GNRI, PNI and CONUT score on prognosis and also because the patients’ ECOG performance status may affect prognosis, we planned a multivariate analysis regarding these 4 parameters.

Multivariate Cox regression analysis was performed to identify independent prognostic factors that could determine PFS. PFS was found to be lower in the GNRI-Low group than in the GNRI-High group [HR (95% CI) = 57.1 (12.8–255.5), (*p* < 0.001)]. In the CONUT-Low group, PFS was found to be higher than in the CONUT- High group [HR (95% CI) = 1.7 (1.0–3.01, *p*: 0.039)]. PFS of the PNI-Low group was shorter than that of the PNI-High group. But it was not significant [HR (95% CI) = 1.8 (0.5–6.2), (*p* = 0.359)]. PFS of those with ECOG Performance score I was longer than PFS of those with ECOG II-III. [HR (95% CI) = 1.1 (0.7–1.9), (*p* < 0.001)]. PFS according to Multivariate analysis is shown in Table 2.

### 3.5 Overall survival analyzes

**Figure 3:**
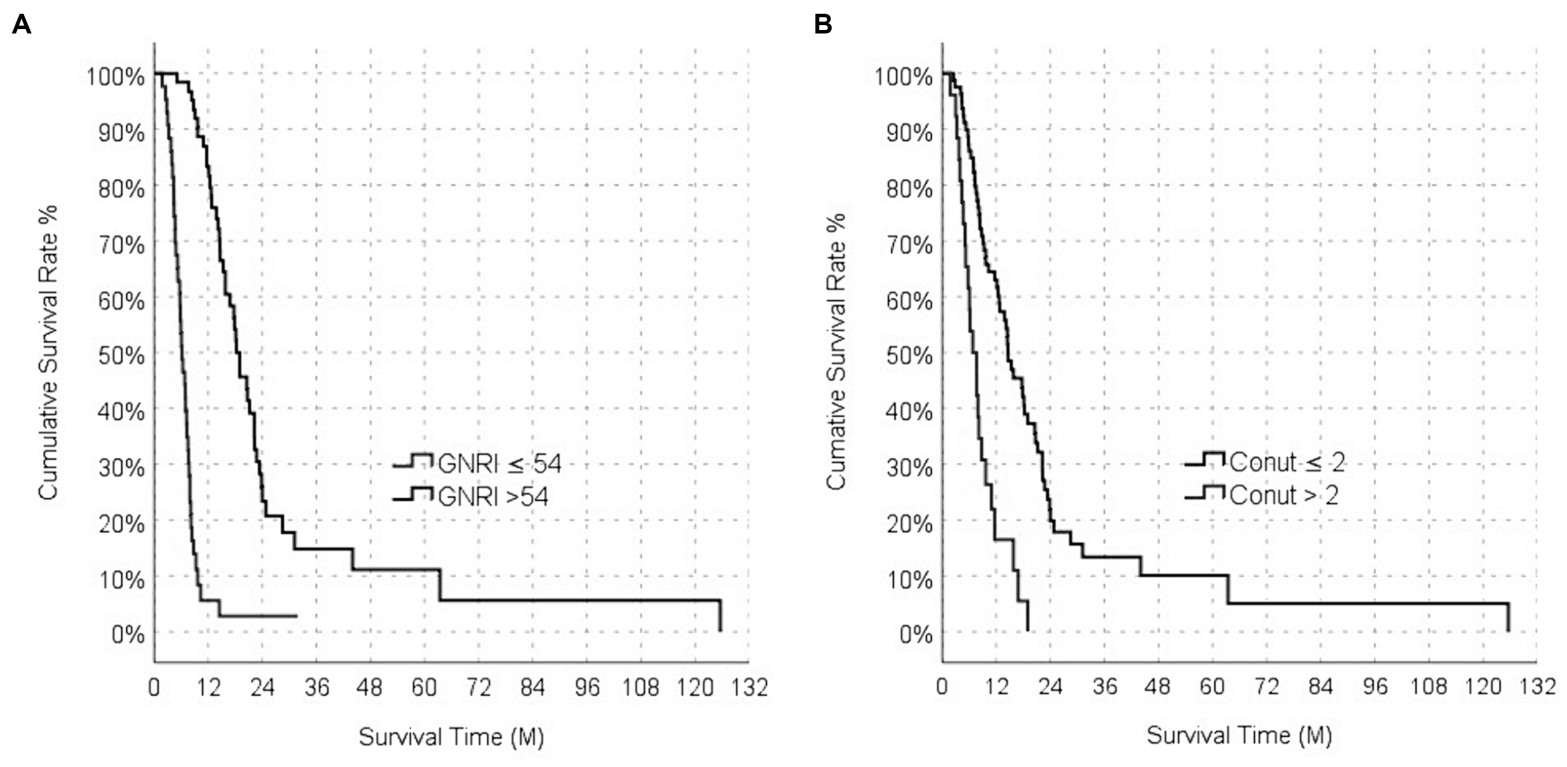
Overall survival analysis functions between GNRI **(A)**, and CONUT **(B)**.

In the entire population, median OS was 8.78 (95% CI: 1.85–16.02) months. The median OS value of the GNRI-High group was 15.9 (95% CI: 1.56–22.03) months, while the median OS value of the GNRI-Low group was 4.89 (95% CI: 0.63–7.38) months. The median OS of the GNRI-High group was significantly longer than that of the GNRI-Low group (*p* < 0.001; Figure 3).

The median OS of the CONUT-Low group was 11.3 (95% CI: 2.3–20.3) months, while the median OS of the CONUT-High group CONUT was 5.1 (95% CI: 0.7–8.0) months. The median OS in the CONUT-Low group was longer than that in the CONUT- High group, and this difference was statistically significant (*p* < 0.001; Figure 3).

The median OS of the PNI-High group was calculated as 15.7 (95% CI: 1.2–20.5) months, while the median OS value of the PNI-Low group was calculated as 5.2 (95% CI: 0.5–6.9) months. The median OS of the PNI-Low group was significantly shorter than that of the PNI-High group (*p* < 0.001).

The median OS was 12.4 (95% CI: 4.8–14.1) months in male patients; it was 7.5 (95% CI: 10.7–18.1) months in female patients. The median OS of male patients was higher than that of female patients and was statistically significant (*p*:0.004). The median OS of those with ECOG Performance score I was 14.7 (95% CI: 10.9–18.4) months while the median OS of those with ECOG II-III was 6.3 (95% CI: 4.2–8.5) months, which was statistically significant (*p* < 0.001). While the median OS of those receiving platinum plus taxane as metastatic first-line chemotherapy was 13.7 (95% CI: 11.1–16.4) months, the median OS of those receiving single-agent gemcitabine was 5.2 (95% CI: 0–12.8) months. The median OS of patients receiving the platinum plus gemcitabine combination was found to be 5.6 (95% CI: 1.4–9.9) months. The median OS of those receiving platinum plus taxane was statistically higher and more significant than the other groups (*p* < 0.001). No significance was found in median OS according to lung, liver and bone metastases. No significance was found in median OS according to diabetes mellitus and hypertension. OS according to univariate analysis is shown in Table 3.

**Table 3:** Univariate and multivariate analysis of OS.

		Multivariate	% 95 HR	*p*	Univariate	% 95 HR		*p*
Overall					12.4	8.8	16.0	
Gender	Female				10.8	7.5	18.2	**0.004**
	Male				12.4	4.9	14.2	
ECOG	≤2			**<0.001**	14.7	10.9	18.5	**<0.001**
	>2	3 (1.7–5.2)			6.3	4.2	8.5	
BMI (Kg/m2)	<18.5				15.3	0.3	36.3	0.730
	18.5–25				12.8	8.1	17.4	
	>25				11.6	8.7	14.6	
First-Line CT								
Platinum + Gemcitabine					5.7	1.4	9.9	**<0.001**
Platinum + Taxane					13.8	11.1	16.4	
Gemcitabine					5.3	0.3	12.9	
Liver metastasis	Yes				12.6	8.3	16.9	0.271
	No				12.4	9.9	14.9	
Lung metastasis	Yes				12.6	8.0	17.2	0.917
	No				11.6	7.9	15.4	
Bone metastasis	Yes				12.6	8.7	16.6	0.742
	No				11.7	7.8	15.6	
HT	Yes				12.6	6.9	18.4	0.591
	No				12.4	8.3	16.6	
DM	Yes				12.6	8.0	17.3	0.191
	No				12.4	6.5	18.3	
GNRI	≤54	12 (3.4–42.6)		**<0.001**	6.1	4.9	7.4	**<0.001**
	>54	Ref			19.0	15.9	22.0	
PNI	≤37	1.9 (0.6–6.8)		0.301	6.0	5.2	6.9	**<0.001**
	>37	Ref			18.1	15.7	20.5	
CONUT	<2	Ref		**0.018**	15.8	11.3	20.3	**<0.001**
	≥2	2 (1.1–3.6)			8.0	0.7	5.1	

BMI, body mass index; CONUT, controlling nutritional status; DM, diabetes mellitus; GNRI, geriatric nutritional risk index; HR, hazard ratio; HT, hypertension; OS, overall survival; PNI, prognostic nutritional index; RT, radiotherapy.

Multivariate Cox regression analysis was performed to identify independent prognostic factors that could determine OS. OS was lower in the GNRI-Low group than in the GNRI-High group [HR (95% CI) = 12 (3.4–42.6), (*p* < 0.001)]. OS was found to be higher in CONUT-Low group than in the CONUT- High group [HR (95% CI) = 2.0 (1.2–3.6), (*p* = 0.018)]. OS of the PNI-Low group was shorter than that of the PNI-High group. But it was not significant [HR (95% CI) = 1.9 (0.6–6.8), (*p* = 0. 301)]. PFS of those with ECOG Performance score I was longer than PFS of those with ECOG II-III. [HR (95% CI) = 3 (1.7–5.2), (*p*: 0.063)]. OS according to multivariate analysis is shown in Table 3.

## 4 Discussion

Many prognostic nutritional factors have been identified in metastatic bladder cancer. Lower body mass index led to increased post-operative complications in patients with operated upper urothelial cancer. Malnutrition seriously affected the prognosis (28). Acar et al., the study revealed that patients with higher HALP scores, which are associated with nutrition, exhibited better survival rates in metastatic bladder cancer. In another study, the correlated prognostic effect of the modified Glasgow prognostic score, which indicates nutritional status, was observed in metastatic bladder cancer (13).

### 4.1 Geriatric nutritional risk index

In this study, we found that the GNRI score are useful prognostic factors in patients with BC aged over 70. Owing to the increasing older adult patients population, special nutritional approaches and prognostic markers have become mandatory. The GNRI, which is a factor that evaluates albumin and desired weight and actual weight, is an important index that combines the two factors and shows acute and chronic problems of nutrition (29).

When we look at the literature, there are many studies on the relationship between GNRI and older cancer. GNRI is a better prognostic factor than the Mini Nutritional Assessment (MNA), previously found in older patients with cancer (30). In a study of 854 patients aged 65 years and older with early-stage cancer, primarily stomach and NSCLC, low GNRI was associated with poor prognosis (31). In a study conducted by Riveros et al. with 1,564 patients aged 65 and over with operated bladder cancer, low GNRI was associated with low survival (19). In a study conducted by Pan et al., which included 442 patients aged 65 and over with bladder cancer who underwent cystectomy, low GNRI was associated with low survival (32).

Many studies have been conducted on urothelial carcinoma in both early and metastatic stages. In a study of 458 patients with upper urinary tract urothelial carcinoma who underwent radical nephrouterectomy, GNRI was found to be an independent predictor of prognosis and postoperative complications (33). In a study of 68 patients with metastatic urothelial cancer receiving cisplatin-gemcitabine in the first row, low GNRI was associated with poor survival (34). In a study evaluating 198 patients with metastatic urothelial carcinoma who received second-line immune checkpoint inhibitors, low GNRI was associated with poor survival (35).

As can be seen, the effect of nutrition on the prognosis of bladder cancer is clear. The prognostic effect of GNRI has been demonstrated in older adult cancer patients. The prognostic effect of GNRI has also been demonstrated in non-bladder cancer and early-stage bladder cancer in patients over the age of 65. To our knowledge, there is no other study other than ours showing the prognostic effect of GNRI in patients with metastatic urothelial cancer over 70.

### 4.2 Controller nutritional status score

Hypoalbuminemia indicates malnutrition. In the presence of malignancy, hypoalbuminemia is observed due to both nutrition and inflammation, and it is known that this leads to a poor prognosis (36). It has been shown that hypercholesterolemia plays a role in cancer pathogenesis and prognosis by causing a decrease in the resistance of cells in tumor growth and metastasis (37). It has been shown that some hematological parameters, especially lymphocyte neutrophils, play an important role in cancer prognosis (38). The CONUT score was developed because these three parameters play a separate role in prognosis (20).

There have been many studies related to this phenomenon in urogenital system. In a study by Claps et al., a high CONUT score was associated with poor PFS in patients with operated bladder cancer (39). In a meta-analysis of 5,040 patients with RCC and upper urothelial carcinoma who underwent nephrectomy, a high CONUT score correlated with poor survival (40). In a study of 347 patients who underwent radical cystectomy, with a median age of 72, it was observed that a high preoperative CONUT score was associated with poor survival (39). In a study of 94 patients with non-invasive BC, the CONUT score obtained before treatment was correlated with poor survival and recurrence (41). In another meta-analysis examining 4,044 patients with metastatic urothelial carcinoma, pretreatment CONUT score was correlated with poor survival (42).

We conducted our study because nutrition affects the prognosis of both bladder cancer and the geriatric population, and we found that a high CONUT score is correlated with a poor prognosis. Our study is consistent with the literature. As far as we know, it is the first study to evaluate the poor prognostic effect of a high CONUT score in patients with metastatic bladder cancer over 70.

High-risk patients can be identified by calculating the GNRI and CONUT score, which affect the prognosis in bladder cancer patients. Thus, if we can identify treatable nutritional factors, starting treatment by correcting them as much as possible before chemotherapy will help improve the prognosis.

### 4.3 Prognostic nutritional index

The PNI is a practical test calculated using serum albumin and lymphocyte counts. Albumin, alone or in combination with other factors, is a negative acute-phase reactant that indicates the nutritional status. Since albumin is a marker regulated by the proinflammatory cytokines IL-1 and IL-6 and Tumor necrosis factor alpha (TNF-alpha), it is associated with inflammation (43). On the other hand, lymphocytes play an important role in cellular immunity by inhibiting the migration of cancer cells, and in the case of lymphocytopenia, the migration and invasion capabilities of cancer cells increase (44).

In the study conducted by Yılmaz et al. in 154 patients with muscle invasive bladder cancer, low PNI was associated with low PFS (45). In the study conducted by Bi et al., which included 387 high-risk NMIBC patients, high PNI predicted higher survival outcomes (46). In a study involving patients with metastatic urothelial carcinoma, a low PNI was associated with poor prognostic factors (47).

The PNI is a prognostic marker for evaluating nutrition and inflammation. We aimed to determine the prognostic effect of PNI in the geriatric population, and found that high PNI led to high survival, but this was not statistically significant. If a study with a homogeneous population with a higher number of patients is planned, PNI may gain prognostic importance.

### 4.4 Strengths and limitations

The fact that recurrent metastatic patients receive treatment until the time of recurrence and different protocols make our population heterogeneous. Considering the high variability of the population and the small sample size, it reduces the power of our analysis.

We acknowledge a limitation of our study in not excluding patients with conditions like familial short stature, obesity, heart failure, and protein-losing nephropathy, which may impact GNRI. Similarly, patients with conditions leading to hypoalbuminemia, such as familial hypercholesterolemia unrelated to nutrition, were not excluded, potentially influencing the CONUT score. Interpretation of these scores should be approached cautiously as they may be influenced by conditions unrelated to nutritional status. A holistic evaluation is recommended to better understand their impact on prognosis.

Although our study is the first to show GNRI and CONUT score as independent predictive factors for PFS and OS in patients in the geriatric population, the retrospective nature of our study was one of the most important limiting factors. Since we could not examine the molecular features of metastatic BC due to technical reasons, the prognosis of the patients may have been affected. Another limiting factor was the lack of clear consensus on cut-off points for the GNRI and CONUT scores due to the limited number of studies. We hope that our study will provide the basis for prospective multicenter studies to better understand the factors that cause survival benefits.

We believe that our study is the first to show that both GNRI and CONUT scores independently serve as prognostic factors for progression-free survival (PFS) and overall survival (OS) in BC patients over 70. However, it is important to note that the retrospective design and the relatively small sample size were the primary constraints of our study.

## 5 Conclusion

In conclusion, the GNRI and CONUT represent practical and successful indices for predicting prognosis in patients over 70 with metastatic BC receiving chemotherapy. If validated through prospective studies, these indices could be useful in routine clinical practice for predicting the prognosis of older patients with metastatic BC.

## Data availability statement

The raw data supporting the conclusions of this article will be made available by the authors, without undue reservation.

## Ethics statement

The studies involving humans were approved by Ethics committee of Diyarbakır Gazi Yaşargil Training and Research Hospital (date of approval and no: 23.6.2023–448). The studies were conducted in accordance with the local legislation and institutional requirements. The participants provided their written informed consent to participate in this study.

## Author contributions

OB: Conceptualization, Investigation, Methodology, Project administration, Resources, Software, Supervision, Visualization, Writing – original draft, Writing – review & editing, Data curation, Formal analysis, Validation. BD: Conceptualization, Data curation, Methodology, Project administration, Resources, Supervision, Writing – review & editing. Yİ: Conceptualization, Data curation, Formal analysis, Investigation, Methodology, Resources, Supervision, Writing – original draft. BA: Conceptualization, Investigation, Methodology, Resources, Supervision, Visualization, Writing – review & editing.
