# Supplementary material for: Geriatric nutritional risk index and controller nutritional status score before metastatic first-line chemotherapy predict survival in patients over 70 years of age with metastatic bladder cancer
Source: Front Med (Lausanne). 2024 May 10;11:1376607. doi: 10.3389/fmed.2024.1376607 (PMC11129015; doi:10.3389/fmed.2024.1376607)
Supplement: Supplementary file 1 [file Table_1.DOCX]

**Supp Table I : Baseline characteristics of the patients according to GNRI**

|  |  | **GNRI ≤ 54 (n:43)** | | | |  | **GNRI >54 (n:63)** | | | | **p** |
| --- | --- | --- | --- | --- | --- | --- | --- | --- | --- | --- | --- |
|  |  | **Mean /n-%** | | | **Median** |  | **Mean /n-%** | | | **Median** |  |
| **Diagnosis Age** |  | 75.0 | ± | 4.0 | 75.0 |  | 75.5 | ± | 4.2 | 75.0 | 0.732 |
| **Gender** | **Male** | 32 |  | 74.4% |  |  | 57 |  | 90.5% |  | ***0.027*** |
|  | **Female** | 11 |  | 25.6% |  |  | 6 |  | 9.5% |  |  |
| **BMI(Kg/m2)** | **<18.6** | 2 |  | 4.7% |  |  | 4 |  | 6.3% |  | 0.710 |
|  | **>18.6** | 41 |  | 95.3% |  |  | 59 |  | 93.7% |  |  |
| **Smoking** | **No** | 10 |  | 23.3% |  |  | 9 |  | 14.3% |  | 0.227 |
|  | **Yes** | 10 |  | 23.3% |  |  | 10 |  | 15.9% |  |  |
|  | **Ex smoker** | 23 |  | 53.5% |  |  | 44 |  | 69.8% |  |  |
| **Additional Disease** | **No** | 10 |  | 23.3% |  |  | 11 |  | 17.5% |  | 0.462 |
|  | **Yes** | 33 |  | 76.7% |  |  | 52 |  | 82.5% |  |  |
| **HT** | **No** | 15 |  | 34.9% |  |  | 26 |  | 41.3% |  | 0.507 |
|  | **Yes** | 28 |  | 65.1% |  |  | 37 |  | 58.7% |  |  |
| **DM** | **No** | 33 |  | 76.7% |  |  | 47 |  | 74.6% |  | 0.801 |
|  | **Yes** | 10 |  | 23.3% |  |  | 16 |  | 25.4% |  |  |
|  | **Yes** | 8 |  | 18.6% |  |  | 15 |  | 23.8% |  |  |
| **ECOG Score** | **0-I** | 25 |  | 58.1% |  |  | 54 |  | 85.7% |  | **<0.001** |
|  | **II** | 15 |  | 34.9% |  |  | 9 |  | 14.3% |  |  |
|  | **III** | 3 |  | 7.0% |  |  | 0 |  | 0.0% |  |  |
| **Histological**  **Type** | **I** | 0 |  | 0.0% |  |  | 1 |  | 1.6% |  | 0.898 |
|  | **II** | 40 |  | 93.0% |  |  | 58 |  | 92.1% |  |  |
|  | **III** | 3 |  | 7.0% |  |  | 4 |  | 6.3% |  |  |
| **Liver**  **Metastasis** | **No** | 40 |  | 93.0% |  |  | 51 |  | 81.0% |  | 0.080 |
|  | **Yes** | 3 |  | 7.0% |  |  | 12 |  | 19.0% |  |  |
| **Lung**  **Metastasis** | **No** | 26 |  | 60.5% |  |  | 34 |  | 54.0% |  | 0.508 |
|  | **Yes** | 17 |  | 39.5% |  |  | 29 |  | 46.0% |  |  |
| **Bone**  **Metastasis** | **No** | 31 |  | 72.1% |  |  | 48 |  | 76.2% |  | 0.634 |
|  | **Yes** | 12 |  | 27.9% |  |  | 15 |  | 23.8% |  |  |
| **Metastatic First-Line CT** | **No** | 0 |  | 0.0% |  |  | 1 |  | 1.6% |  | 1.000 |
|  | **Yes** | 43 |  | 100.0% |  |  | 62 |  | 98.4% |  |  |
| **Exitus** | **No** | 2 |  | 4.7% |  |  | 18 |  | 28.6% |  | **0.002** |
|  | **Yes** | 41 |  | 95.3% |  |  | 45 |  | 71.4% |  |  |
|  |  |  |  |  |  |  |  |  |  |  |  |

BMI: body mass index, CT: Chemotherapy, DM: diabetes mellitus, GNRI: geriatric nutritional risk index, HT:hypertension, RT: radiotherapy.

**Supp Table II : Baseline characteristics of the patients according to CONUT**

|  |  | **Low-CONUT (n:80)** | | | | **High-CONUT (n:26)** | | | | ***p*** |
| --- | --- | --- | --- | --- | --- | --- | --- | --- | --- | --- |
|  |  | **Mean /n-%** | | | **Median** | **Mean /n-%** | | | **Median** |  |
| **Diagnosis Age** | | 75.4 | ± | 4.2 | 75.0 | 75.0 | ± | 3.9 | 75.0 | 0.871 |
| **Gender** | Male | 66 |  | 82.5% |  | 23 |  | 88.5% |  | 0.472 |
|  | Female | 14 |  | 17.5% |  | 3 |  | 11.5% |  |  |
| **BMI(Kg/m2)** | <18.6 | 5 |  | 6.3% |  | 1 |  | 3.8% |  | 1.000 |
|  | >18.6 | 75 |  | 93.8% |  | 25 |  | 96.2% |  |  |
| **Smoking** | No | 15 |  | 18.8% |  | 4 |  | 15.4% |  | 0.478 |
|  | Yes | 13 |  | 16.3% |  | 7 |  | 26.9% |  |  |
|  | Ex smoker | 52 |  | 65.0% |  | 15 |  | 57.7% |  |  |
| **Additional Disease** | No | 15 |  | 18.8% |  | 6 |  | 23.1% |  | 0.631 |
|  | Yes | 65 |  | 81.3% |  | 20 |  | 76.9% |  |  |
| **HT** | No | 32 |  | 40.0% |  | 9 |  | 34.6% |  | 0.624 |
|  | Yes | 48 |  | 60.0% |  | 17 |  | 65.4% |  |  |
| **DM** | No | 62 |  | 77.5% |  | 18 |  | 69.2% |  | 0.395 |
|  | Yes | 18 |  | 22.5% |  | 8 |  | 30.8% |  |  |
|  | Yes | 16 |  | 20.0% |  | 7 |  | 26.9% |  |  |
| **ECOG Score** | 0-I | 65 |  | 81.3% |  | 14 |  | 53.8% |  | ***0.005*** |
|  | II | 14 |  | 17.5% |  | 10 |  | 38.5% |  |  |
|  | III | 1 |  | 1.3% |  | 2 |  | 7.7% |  |  |
|  | No | 53 |  | 88.3% |  | 44 |  | 95.7% |  |  |
| **Histological Type** | I | 1 |  | 1.3% |  | 0 |  | 0.0% |  | 1.000 |
|  | II | 74 |  | 92.5% |  | 24 |  | 92.3% |  |  |
|  | III | 5 |  | 6.3% |  | 2 |  | 7.7% |  |  |
| **Liver Metastasis** | No | 69 |  | 86.3% |  | 22 |  | 84.6% |  | 0.835 |
|  | Yes | 11 |  | 13.8% |  | 4 |  | 15.4% |  |  |
| **Lung Metastasis** | No | 44 |  | 55.0% |  | 16 |  | 61.5% |  | 0.559 |
|  | Yes | 36 |  | 45.0% |  | 10 |  | 38.5% |  |  |
| **Bone Metastasis** | No | 61 |  | 76.3% |  | 18 |  | 69.2% |  | 0.475 |
|  | Yes | 19 |  | 23.8% |  | 8 |  | 30.8% |  |  |
| **Metastatic First-Line CT** | No | 1 |  | 1.3% |  | 0 |  | 0.0% |  | 1.000 |
|  | Yes | 79 |  | 98.8% |  | 26 |  | 100.0% |  |  |
| **Exitus** | No | 18 |  | 22.5% |  | 2 |  | 7.7% |  | 0.094 |
|  | Yes | 62 |  | 77.5% |  | 24 |  | 92.3% |  |  |

BMI: body mass index , CT: Chemotherapy, CONUT: controlling nutritional status, DM: diabetes mellitus, HT: hypertension, RT: radiotherapy

**Supp Table III: Baseline characteristics of the patients according to PNI**

|  |  | **PNI ≤ 37 (n:40)** | | | | **PNI > 37 (n:66)** | | | | ***p*** | |
| --- | --- | --- | --- | --- | --- | --- | --- | --- | --- | --- | --- |
|  |  | **Mean /n-%** | | | **Median** | **Mean /n-%** | | | **Median** |  |  |
| **Diagnosis Age** | | 75.3 | ± | 4.0 | 75.0 | 75.3 | ± | 4.1 | 74.5 | 0.842 |  |
| **Gender** | Male | 29 |  | 72.5% |  | 60 |  | 90.9% |  | ***0.012*** |  |
|  | Female | 11 |  | 27.5% |  | 6 |  | 9.1% |  |  |  |
| **BMI(Kg/m2)** | <18.6 | 1 |  | 2.5% |  | 5 |  | 7.6% |  | 0.273 |  |
|  | >18.6 | 39 |  | 97.5% |  | 61 |  | 92.4% |  |  |  |
| **Smoking** | No | 10 |  | 25.0% |  | 9 |  | 13.6% |  | 0.182 |  |
|  | Yes | 9 |  | 22.5% |  | 11 |  | 16.7% |  |  |  |
|  | Ex smoker | 21 |  | 52.5% |  | 46 |  | 69.7% |  |  |  |
| **Additional Disease** | No | 9 |  | 22.5% |  | 12 |  | 18.2% |  | 0.589 |  |
|  | Yes | 31 |  | 77.5% |  | 54 |  | 81.8% |  |  |  |
| **HT** | No | 13 |  | 32.5% |  | 28 |  | 42.4% |  | 0.309 |  |
|  | Yes | 27 |  | 67.5% |  | 38 |  | 57.6% |  |  |  |
| **DM** | No | 30 |  | 75.0% |  | 50 |  | 75.8% |  | 0.930 |  |
|  | Yes | 10 |  | 25.0% |  | 16 |  | 24.2% |  |  |  |
|  | Yes | 7 |  | 17.5% |  | 16 |  | 24.2% |  |  |  |
| **ECOG Score** | 0-I | 22 |  | 55.0% |  | 57 |  | 86.4% |  | ***<0.001*** |  |
|  | II | 15 |  | 37.5% |  | 9 |  | 13.6% |  |  |  |
|  | III | 3 |  | 7.5% |  | 0 |  | 0.0% |  |  |  |
| **Liver Metastasis** | No | 54 |  | 92.5% |  | 54 |  | 81.8% |  | 0.126 |  |
|  | Yes | 12 |  | 7.5% |  | 12 |  | 18.2% |  |  |  |
| **Lung Metastasis** | No | 36 |  | 60.0% |  | 36 |  | 54.5% |  | 0.583 |  |
|  | Yes | 30 |  | 40.0% |  | 30 |  | 45.5% |  |  |  |
| **Bone Metastasis** | No | 50 |  | 72.5% |  | 50 |  | 75.8% |  | 0.709 |  |
|  | Yes | 16 |  | 27.5% |  | 16 |  | 24.2% |  |  |  |
| **Metastatic First-Line CT** | No | 1 |  | 0.0% |  | 1 |  | 1.5% |  | 1.000 |  |
|  | Yes | 65 |  | 100.0% |  | 65 |  | 98.5% |  |  |  |
| **Exitus** | No | 18 |  | 5.0% |  | 18 |  | 27.3% |  | ***0.004*** |  |
|  | Yes | 48 |  | 95.0% |  | 48 |  | 72.7% |  |  |  |

BMI: body mass index, CT: Chemotherapy, DM: diabetes mellitus, HT:hypertension, PNI: prognostic nutritional index; RT: radiotherapy
